# Molecular Mechanism Underlying Persistent Induction of LCN2 by Lipopolysaccharide in Kidney Fibroblasts

**DOI:** 10.1371/journal.pone.0034633

**Published:** 2012-04-13

**Authors:** Trevor Glaros, Yan Fu, Jianhua Xing, Liwu Li

**Affiliations:** 1 Laboratory of Innate Immunity and Inflammation, Virginia Polytechnic Institute and State University, Blacksburg, Virginia, United States of America; 2 Department of Biological Science, Virginia Polytechnic Institute and State University, Blacksburg, Virginia, United States of America; 3 Interdisciplinary Program of Genetics, Bioinformatics and Computational Biology, Virginia Polytechnic Institute and State University, Blacksburg, Virginia, United States of America; University of Southern California, United States of America

## Abstract

The neutrophil gelatinase-associated lipocalin 2 (LCN2) is a critical inflammatory mediator persistently induced during endotoxemia, contributing to tubular damage and kidney failure. The intracellular process responsible for persistent induction of LCN2 by bacterial endotoxin Lipopolysaccharide (LPS) is not well understood. Using primary kidney fibroblasts, we observed that LPS-induced LCN2 expression requires a coupled circuit involving an early transient phase of AP-1 path and a late persistent phase of C/EBPδ path, both of which are dependent upon the interleukin 1 receptor associated kinase 1 (IRAK-1). Using immunoprecipitation analysis we observed transient binding of AP-1 to the promoters of both *TNFα* and *C/ebpδ*. On the other hand, we only observed persistent binding of C/EBPδ to its own promoter but not on *TNFα*. Blockage of new protein synthesis using cyclohexamide significantly reduced the expression of C/EBPδ as well as LCN2. By chromatin immunoprecipitation analyses, we demonstrated that LPS recruited C/EBPδ to the *Lcn2* promoter in WT, but not IRAK-1 deficient fibroblasts. A differential equation-based computational model captured the dynamic circuit leading to the persistent induction of LCN2. In vivo, we observed elevated levels of LCN2 in kidneys harvested from LPS-injected WT mice as compared to IRAK-1 deficient mice. Taken together, this study has identified an integrated intracellular network involved in the persistent induction of LCN2 by LPS.

## Introduction

Initially identified in neutrophils as a gelatinase-associated small protein [1], LCN2 was recognized as an innate defense molecule by sequestering and depleting iron-containing siderophores and curbing bacterial growth [2]. Recent studies reveal that LCN2 can be potently induced by inflammatory stimulants and widely expressed in vital organs and tissues such as kidney, heart, and brain [3], [4], [5], [6]. Elevated level of LCN2 in kidney is a well-recognized marker for both chronic kidney diseases, reflecting the extent of kidney damage [7], [8]. LCN2 is not only a marker, but also a key contributor of kidney disease, as reflected by the alleviation of chronic kidney damage in mice with *Lcn2* gene deletion [6].

Bacterial endotoxin (lipopolysaccharide-LPS) is a potent inducer of LCN2 [9], [10], [11]. Elevated levels of endotoxin are seen in both acute and chronic conditions. Acute endotoxemia leads to septic shock and multi-organ damages including acute kidney failure [12]. In contrast, chronic endotoxemia associated with obesity, aging, and other adverse health conditions can lead to persistent inflammatory complications and chronic diseases [11], [13], [14]. LCN2 levels persist during both acute and chronic phases of kidney damage, and may serve as a key propagating factor for chronic kidney disease [6]. However, the molecular mechanism underlying the persistent expression of LCN2 is not well understood.

Studies using other inflammatory stimulants such as IL-17 and IL-1β indicate that the induction of LCN2 requires multiple transcription factors including AP-1 and C/EBPδ [9], [15]. AP-1 activation by LPS is transient and responsible for the transient induction of pro-inflammatory mediators [16]. On a separate report, C/EBPδ was implicated in propagating the persistent activation of TLR4 pathway [17].

Based on these observations, we examined the molecular circuit underlying the persistent induction of LCN2 by LPS in kidney fibroblasts. We identified that IRAK-1, a key TLR4 intracellular signaling component, is involved in coordinating both the transient activation of AP-1, as well as the persistent activation of C/EBPδ, which are collectively responsible for the sustained expression of LCN2. We further performed a differential equation-based computational modeling, which complemented our experimental data and revealed a dynamic intracellular signaling circuit responsible for the persistent induction of LCN2 by LPS.

## Materials and Methods

### Reagents

LPS (*Escherichia coli* 0111:B4) and Cycloheximide were obtained from Sigma Aldrich. Anti-LCN2 antibody was purchased from R&D Systems. Anti- IkBα antibody was purchased from Cell Signaling. Anti-Lamin B (ab-16048) was purchased from Abcam. Anti-C/EBPδ (M-17), anti-cJun (H-79), anti-P65 (F-6), anti-GAPDH (FL-335), anti-IRAK-1 (F-4), and anti-β-actin (C-4) antibodies were from Santa Cruz Biotechnology.

### Kidney Fibroblast cultures

Both WT and IRAK-1^−/−^ C57/BL/6 mice were housed and bred in the Derring Hall Animal Facility in compliance with approved Animal Care and Use Committee protocols at Virginia Polytechnic Institute and State University. Murine kidney fibroblasts were isolated as previously described [18]. Fibroblast cells were grown in 50∶50 DMEM/F12 #10-092-CV (Mediatech™, Inc., VA) supplemented with 10% FBS #SH30071.03 (Hyclone™) and 1% Penicillin/Streptomycin at 37°C with 5% CO_2_.

### Real time RT-PCR (qRT-PCR)

Total RNAs were harvested from murine kidney fibroblasts treated with or without LPS using Trizol® Reagent (Invitrogen) according to the manufacturer's protocol. cDNAs were generated using the High Capacity cDNA Reverse Transcription Kit (Applied BioSystems™, Foster, CA). Real-time PCR analyses were performed using the iQ Sybr® Green Supermix (BioRad™ Laboratories, Hercules, CA) on an IQ5 thermocycler (BioRad). The relative levels of transcripts were calculated using the ΔΔCt method using *GAPDH* as the internal control. The relative levels of mRNA from the untreated samples were adjusted to 1 and served as the basal control value. The following primers were used perform qRT-PCR: mouse *LCN2* forward: 5′- TTT CAC CCG CTT TGC CAA GT-3′, reverse: 5′-GTC TCT GCG CAT CCC AGT CA-3′; mouse *C/ebpδ* forward: 5′-ACT TCA GCG CCT ACA TTG ACT CCA-3′, reverse: 5′-TGT TGA AGA GGT CGG CGA AGA GTT-3′; mouse *GAPDH* forward: 5′-AAC TTT GGC ATT GTG GAA GGG CTC-3′, reverse: 5′- GGA AGA GTG GGA GTT GCT GTT GA-3′; mouse *TNFα* forward: 5′-AGC CGA TGG GTT GTA CCT TGT CTA-3′, reverse: 5′-TGA GAT AGC AAA TCG GCT GAC GGT-3′; mouse *Il-6* forward: 5′-ATC CAG TTG CCT TCT TGG GAC TGA-3′, reverse: 5′- TAA GCC TCC GAC TTG TGA AGT GGT-3′; mouse *SOCS1* forward: 5′-AGT CGC CAA CGG AAC TGC TTC TT-3′, reverse: 5′-ACG TAG TGC TCC AGC AGC TCG AAA-3′; and mouse *ATF3* forward: 5′-TCA AGG AAG AGC TGA GAT TCG CCA-3′, reverse: 5′-GTT TCG ACA CTT GGC AGC AGC AAT-3′.

### Western Blot Analysis

Isolation of whole cell, cytoplasmic, and nuclear lysates were performed as described previously [19]. Briefly, fibroblasts were lysed on ice using lysis buffer (10 mM HEPES, pH 7.9, 1.5 mM MgCl2, 10% Triton X-100, 10 mM KCl, 0.5 mM EDTA, 0.5 mM dithiothreitol, 0.5 mM phenylmethylsulfonyl fluoride, 1 ug/mL leupeptin, 1 ug/mL pepstatin) for 30 minutes. Samples were centrifuged at 5000 rpm for 10 minutes at 4°C. The supernatants were saved as the cytoplasmic extract and the pelleted nuclei were re-suspend and lysed in a high salt buffer (20 mM HEPES, pH 7.9, 1.5 mM MgCl2, 0.4 M NaCl, 0.2 mM EDTA, 0.5 mM dithiothreitol, 1 mM phenylmethylsulfonyl fluoride) on ice for 30 minutes. The samples were then centrifuged at 12,000 RPM for 20 minutes at 4°C and supernatant kept as the nuclear extract. Protein samples were analyzed by Western Blot as described before. Images were quantified with Fujifilm Multi Gauge software normalized against β actin, GAPDH, or Lamin B.

### Chromatin Immunoprecipitation (ChIP) assays

Protein and chromatin from treated cells were cross-linked using 1% formaldehyde solution in complete media for 15 minutes with gentle rocking every 3 minutes at room temperature. Cells were washed twice with cold PBS and treated with a glycine solution for 5 minutes to stop the cross-linking reaction. The cells were then lysed in an ice cold buffer containing SDS and protease inhibitor cocktail. The samples were then subject to sonication in an ice water bath to shear the chromatin for 6 minutes (30 seconds on and 30 seconds off). The sheared chromatin was processed using Chip-IT Express Kit #53008 from Active Motif™. The immune-precipitated chromatin was analyzed by PCR using primer pairs that span the promoter regions of either *Lcn2*, *C/ebpδ, and Tnfα*. The primer sequences are as follows: mouse p*Lcn2* forward: 5′-TGA CCC ACA AGC AGT GCC CTG T-3′, reverse: 5′-ACT TGG CAA GAT TTC TGT CCC-3′; mouse p*C/ebpδ* forward: 5′-ACA AAC AGG AAG GAG GGA AG-3′, reverse: 5′-TCC AAG TTG GGC TGT CA-3′; and mouse p*Tnfα* forward: 5′-AGG AGA TTC CTT GAT GCC TGG GT-3′, reverse: 5′-TTT CTG TTC TCC CTC CTG GCT AGT-3′.

### In vivo LPS injection

Female IRAK-1^−/−^ and WT C57/BL/6 mice of 12 weeks old received intraperitoneal injections with 25 mg/kg of LPS. Six hours after the injection, mice were sacrificed and the whole kidney tissue was harvested for subsequent mRNA analysis by real time RT-PCR and protein analyses by Western blot.

### Computational analysis

A differential-equation based computational modeling is performed as previously described [20]. A detailed description of the modeling approach is provided in the supplementary document accompanying this manuscript.

### Statistical Analysis

Data significance was determined using the student t-test. P-values less than 0.05 were considered statistically significant as indicated by an asterisk (*).

## Results

### LPS induces persistent expression of LCN2 in kidney fibroblasts in an IRAK-1 dependent fashion

**Figure 1:**
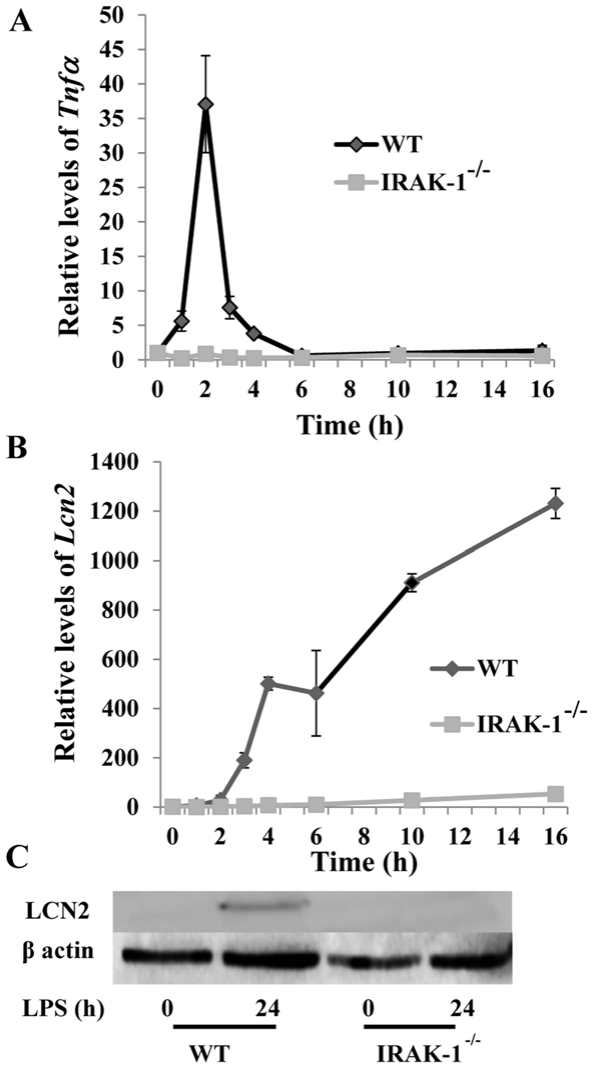
LPS stimulation induces a persistent induction of LCN2 in kidney fibroblasts. (A) LPS (100 ng/ml) induces a transient induction of *Tnfα* mRNA. (B) LPS (100 ng/ml) induces a persistent induction of *Lcn2* mRNA Transcript levels were measured by qRT-PCR as described above. The results are expressed as means +/− standard deviation performed in triplicate. (C) LCN2 protein levels persist after 24 hours of LPS stimulation. The levels of LCN2 were visualized by western blot.

We first compared the expression profiles of selected inflammatory genes in fibroblasts stimulated with LPS. As shown in Figure 1A, the induction of *Tnfα* message by LPS was transient, peaked at the 2 hour time point post challenge, and returned to basal values at the 16 hour time point. In contrast, the expression of *Lcn2* mRNA persisted throughout the course of LPS treatment (Fig. 1B). Because IRAK-1 is one of the key downstream components of TLR4 pathway, we evaluated whether IRAK-1 is involved in both the transient induction of *Tnfα* and persistent induction of *Lcn2* message. As shown in Figure 1A and 1B, the inductions of both *Tnfα* and *Lcn2* were significantly reduced in IRAK-1 deficient kidney fibroblasts. We also confirmed the induction of LCN2 protein in wild type (WT) cells by LPS (Fig. 1C).

### LPS induces transient activation of AP-1 and persistent induction of C/EBPδ in kidney fibroblasts

**Figure 2:**
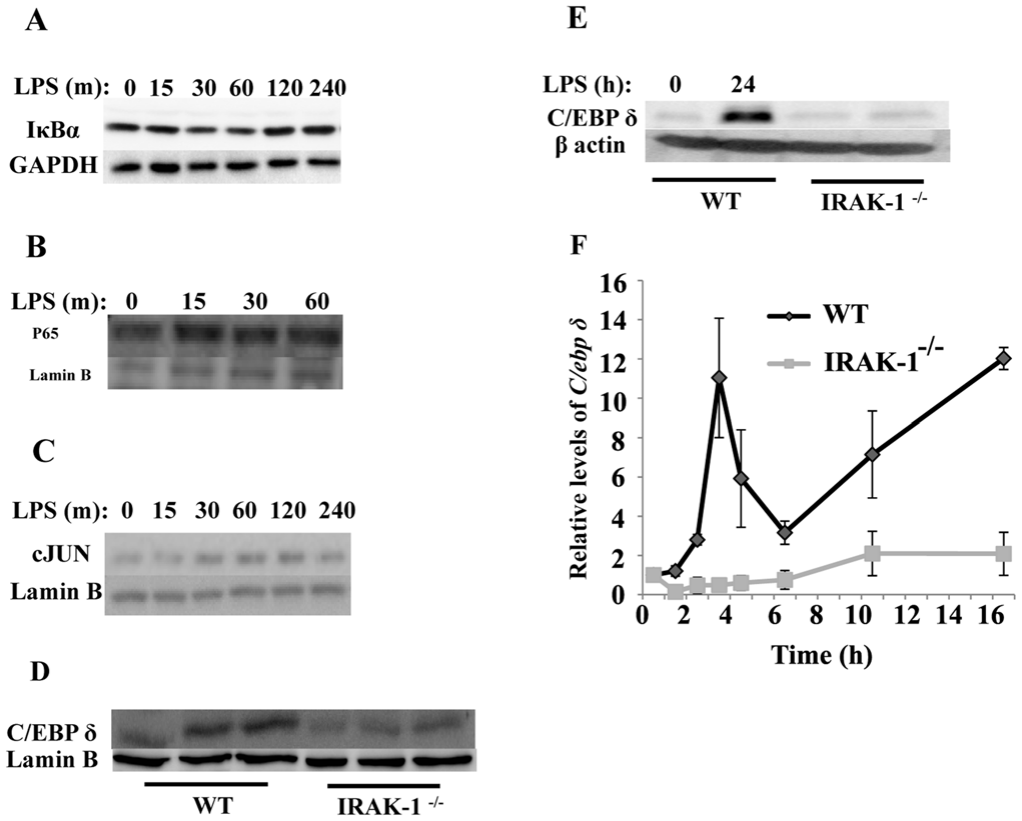
LPS induces transient activation of AP-1 and persistent induction of C/EBPδ in kidney fibroblasts. WT fibroblasts were treated with LPS (100 ng/ml) for the indicated time periods. (A) LPS fails to significantly induce IκBα degradation. Whole cell lysates were harvested and analyzed by Western blot using an IκBα specific antibody. (B) LPS fails to significantly induce p65 nuclear translocation in kidney fibroblasts. Nuclear cell lysates were harvested and analyzed by Western blot using an anti-p65 antibody. (C) LPS induces the active translocation of AP-1/c-Jun. Nuclear lysates were harvested and analyzed by Western blot. (D&E) LPS induces a persistent increase of C/EBPδ protein. Whole cell lysates were harvested and analyzed by Western blot. (F) LPS significantly induces the expression of *C/ebpδ* dependent upon IRAK-1. WT and IRAK-1 deficient cells were treated with LPS for indicated time period. Message levels of *C/ebpδ* were determined by RT-PCR. n = 3; *P<0.05.

To determine the potential mechanism for the persistent induction of LCN2 in kidney fibroblasts by LPS, we examined the activation status of relevant transcription factors including NFκB, AP-1 and C/EBPδ. As shown in Figure 2A, LPS treatment caused no apparent decrease in the levels of Iκbα in WT fibroblasts, indicating a lack of robust NFκB activation by LPS in kidney fibroblasts. Further supporting this claim, we observed that the residue nuclear p65/RelA remained constant following LPS treatment (Fig. 2B). In contrast, we observed a rapid yet transient induction of AP-1/c-Jun. Nuclear levels of AP-1/c-Jun was induced by LPS, reached its peak level by 60 to 120 minutes, and decreased at 240 minutes after LPS challenge (Fig. 2C). C/EBPδ, on the other hand, was elevated after 24 hr of LPS treatment (Fig. 2D and 2E). Given the fact that Western blot may not give rise to quantitative result, we performed RT-PCR analysis of *C/ebpδ* expression induced by LPS. As shown in Figure 2F, LPS significantly induced the expression of *C/ebpδ*, and such induction is dependent on IRAK-1.

**Figure 3:**
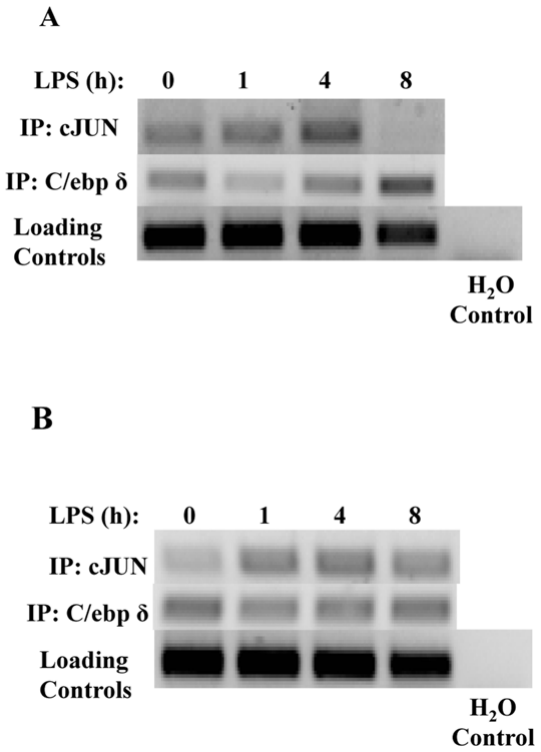
LPS induces transient recruitment of c-Jun to the promoters of *Tnfα* and *C/ebpδ*, and induces prolonged recruitment of C/EBPδ to the promoter of *C/ebpδ*. WT kidney fibroblasts were treated with or without LPS for a time course. Nuclear lysates were then subjected to ChIP analysis to examine relative binding to the promoter of *C/ebpδ* (A) or *Tnfα* (B).

Based on above observation and previous studies, we propose that a coupled step-wise process may lead to the persistent activation and induction of C/EBPδ. LPS may first induce an initial C/EPBδ expression primarily through a transient activation of AP-1 and residue NFκB in the nucleus [21]. Subsequently, newly synthesized C/EBPδ can auto-induce its own expression and contribute to a steady induction of C/EBPδ [17] and its downstream target gene LCN2. To test this hypothesis, we performed Chromatin immunoprecipitation (ChIP) analysis to determine the recruitment of c-Jun and C/EBPδ to the promoter of *C/ebpδ*. As shown in Figure 3, LPS transiently increased the recruitment of c-Jun to the promoter of *C/ebpδ* (Figure 3A) and *Tnfα* (Figure 3B), peaking at 4 hr for *C/ebpδ* and 1 hr for *Tnfα* promoter, and dropping off at the 8 hr time point. In contrast, increased recruitment of C/EBPδ at the 8 hr time point was evident on the promoter of *C/ebpδ*, but not *Tnfα*.

### Both *De novo* synthesis and subsequent activation of C/EBPδ is required for the expression of LCN2

**Figure 4:**
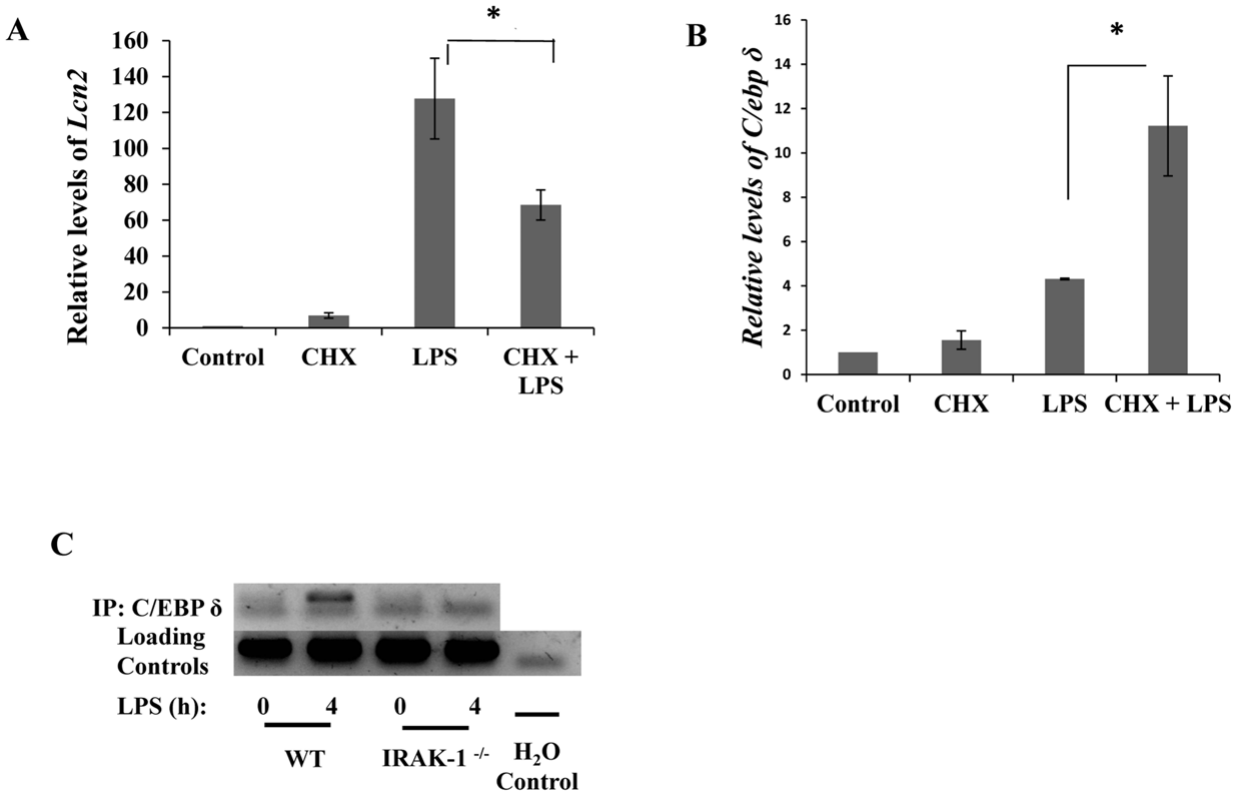
*Lcn2* transcription requires new protein synthesis, but *C/ebpδ* does not. C/EBPδ is recruited to the promoter of *LCN2* upon LPS stimulation in an IRAK-1 dependent manner. (A) Cyclohexamide, a protein synthesis inhibitor, blocks *Lcn2* transcription upon LPS stimulation. (B) The induction of *C/ebpδ* does not require new protein synthesis. (B) Wild-type kidney fibroblasts were untreated (DMSO) or pretreated with cyclohexamide 5 ug/mL for 1 hour. Immediately following pretreatment, the cells were stimulated with or without LPS for 4 hours. Transcripts were quantitated using qRT-PCR and standardized using *Gapdh* as the internal loading control. Each experiment was performed in triplicate. *P<0.05 (C) The recruitment of C/EBPδ to the promoter of LCN2 upon LPS stimulation depends upon IRAK-1. Nuclear lysates were subject to ChIP analysis using a C/EBPδ specific antibody. The arrow points to the specific amplification signal for *C/ebpδ* promoter. The * sign indicates a non-specific amplification.

To further confirm that *de novo* synthesis of C/EBPδ is involved in LPS-induced LCN2 expression, we applied cycloheximide (CHX), a known protein synthesis inhibitor, to the culture medium. As shown in Figure 4A, CHX significantly reduced LPS-mediated induction of *Lcn2* message, suggesting that new protein synthesis is required for the induction of LCN2 gene expression. In contrast, CHX has no inhibitory effect on LPS-mediated expression of *C/ebpδ* message (Figure 4B). This is in agreement with a previous finding that the expression of C/EBPδ and its subsequent transcriptional activation are two separate events controlled by two distinct upstream processes [21]. To further confirm the role of C/EBPδ in LPS induced expression of *Lcn2*, we examined the recruitment of C/EBPδ to the promoter of *Lcn2* following LPS challenge. As shown in Figure 4C, LPS dramatically prolonged the elevated recruitment of C/EBPδ to the *Lcn2* promoter in WT cells.

### The lack of inducible negative regulators contributes to prolonged expression of LCN2 in kidney fibroblasts upon LPS stimulation

**Figure 5:**
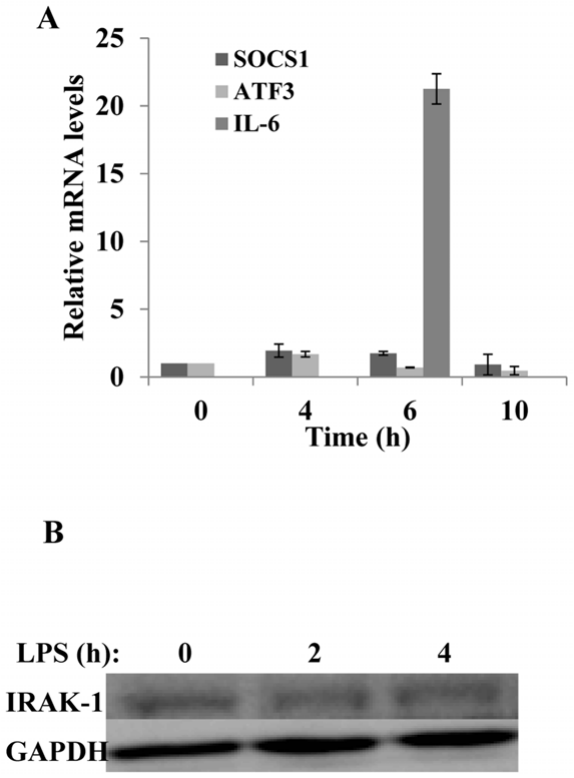
*Socs1* and *Atf3*, negative regulators of TLR4 signaling, are not induced and IRAK-1 remains intact upon LPS stimulation in kidney fibroblasts. (A) The expressions of *Socs1* and *Atf3* were not induced after stimulation with LPS. Wild-type kidney fibroblasts were either untreated or treated with 100 ng/mL LPS for 4, 6, or 10 hours. *Socs1*, *Atf3*, and *Il-6* transcripts were measured by real time RT-PCR assays and standardized against *Gapdh* levels. Each experiment was performed in triplicate. Data is depicted as means +/− standard deviation. (B) LPS does not cause the degradation of IRAK-1. Wild-type kidney fibroblasts were either untreated or treated with 100 ng/mL LPS for either 2 or 4 hours. Whole cell lysates were harvested and analyzed by Western blot with IRAK-1 specific antibodies.

Through the classical NFκB pathway, LPS is known to induce negative regulators that subsequently turn off the expression of pro-inflammatory genes. Among the negative regulators, SOCS1 suppresses the upstream LPS signaling pathway by inhibiting IRAK-1 [22]. On the other hand, ATF3 is shown to compete and suppress the activity of C/EBPδ [17], [23]. Given the fact that we did not observe noticeable activation of the NFκB pathway, we hypothesize that LPS may not be able to induce related negative regulators in kidney fibroblasts. Indeed, we demonstrated that LPS failed to induce the expression of *Socs 1* and *Atf 3* (Fig. 5A). As a consequence, we observed that there was no apparent degradation of IRAK-1 protein in kidney fibroblasts following a 4 hr LPS treatment (Fig. 5B). Taken together, our data indicate that LPS treatment in kidney fibroblasts failed to induce negative regulators that modulate the persistent activation of C/EBPδ, leading to a sustained induction of its downstream gene *Lcn2*.

### In vivo induction of LCN2 expression in an IRAK-1 dependent fashion

**Figure 6:**
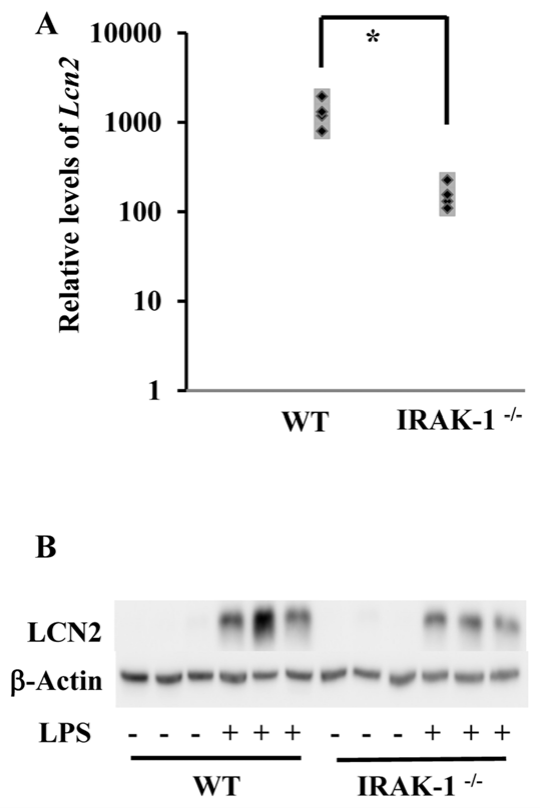
Loss of IRAK-1 causes decreased expression of *Lcn2* mRNA and protein *in vivo*. WT and IRAK-1^−/−^ C57/BL/6 female mice of 12 weeks old (6 each) were intraperitoneally injected with either 30 mg/kg of LPS or PBS for 6 hours. (A) Kidney tissues were extracted and subject to RNA extraction. *Lcn2* transcripts were measured by qRT-PCR assays and standardized against their respective controls (mice receiving PBS injections). Data is depicted as three separate mice (both WT and IRAK-1^−/−^). *P<0.05 (B) Protein lysates were extracted from the kidney tissues and subjected to Western blot. Blots were analyzed using LCN2 specific antibodies. The same blot was probed with β-actin as a loading control.

To test the *in vivo* relevance of our finding, we examined the expression of LCN2 in kidney tissues from WT and IRAK-1 deficient mice following LPS challenge. As shown in Figure 6, LPS injection induced significant induction of *Lcn2* messages as well as LCN2 proteins in kidney tissues harvested from LPS-injected WT mice. In contrast, the levels of *Lcn2* messages were significantly lower in kidneys harvested from IRAK-1 deficient mice injected with LPS.

### Computational analyses of the molecular circuit responsible for the persistent induction of LCN2 in kidney fibroblasts

**Figure 7:**
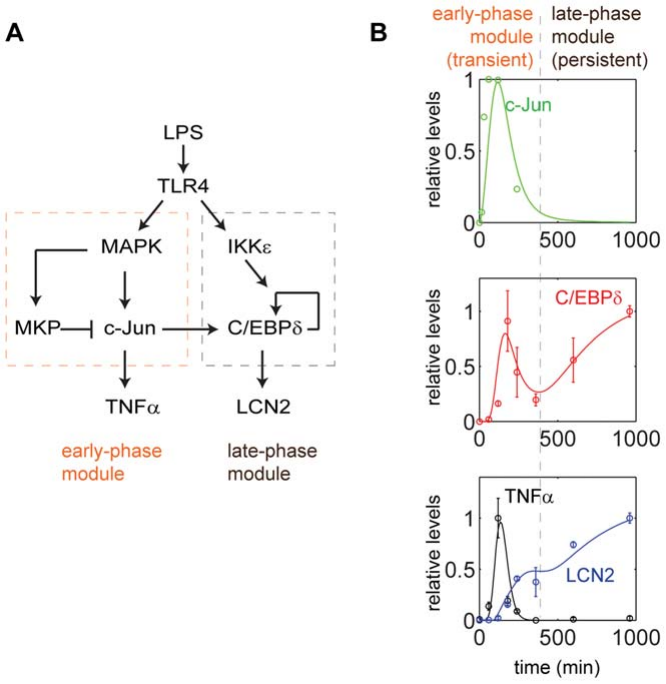
Computational simulation captures the dynamic features of the experimental data. (A) The network motif responsible for the transient induction of *Tnfα* and the persistent induction of *Lcn2*. (B) Computational simulation of the transient induction of *Tnfα* and persistent induction of *Lcn2*. Circles denote actual experimental data. Lines represent computational simulations.

We further fit our experimental data into a differential equation based computation model (Fig. 7). This model takes into consideration of the positive feedback loop involving C/EBPδ that helps to maintain a sustained expression of LCN2.

This model is based on a coarse-grained TLR4-signaling network that highlights the core pathways controlling the induction of LCN2. As is shown in Figure 7A, this signaling circuit is composed of both an early-phase module with transient response and a late-phase module with persistent response. Each module can be viewed as an information processor that receives inputs from upstream signals, and generate outputs that regulate downstream response. The two modules are connected. The transcription of C/EBPδ is dependent on AP-1/c-Jun, and serves as the core of the late-phase module.

Table 1 lists the equations in the computational model. Each ODE describes the change of a molecular species in the relative level as a function of time. Parameters such as mRNA degradation rates are estimated based on our data or from existing literatures. Other parameters such as activating threshold k and relative weights K are estimated using genetic algorithm of least square regression (MATLAB® 2009b, the MathWorks, Natick, MI). All parameters are listed in Table 2.

**Table 1:** Ordinary Differential Equations used in the model.

Variable	Name	Differential Equations
*x*_1_	c-Jun	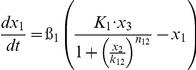
*x*_2_	MKP-1	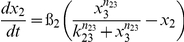
*x*_3_	MAPK	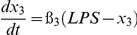
*x*_4_	TNFα	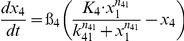
*x*_5_	IKKε	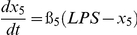
*x*_6_	C/EBPδ	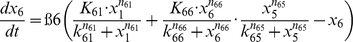
*x*_7_	LCN2	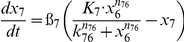

Subscripts of parameters denote the direction of regulations. For example, *k*_61_ represents the threshold for c-Jun (x_1_) to activate C/EBPδ (x_6_).

**Table 2:** List of the estimated parameter values.

Parameter	Value	Description
*β*_1_	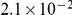	Degradation rate of *x*_1_ (based on Western Blot data)
*β*_2_	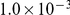	Degradation rate of *x*_2_
*β*_3_	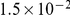	Degradation rate of *x*_3_
*β*_4_	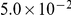	Degradation rate of *x*_4_ (based on RT-PCR data)
*β*_5_	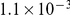	Degradation rate of *x*_5_
*β*_6_	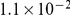	Degradation rate of *x*_6_, based on (2)
*β*_7_	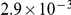	Degradation rate of *x*_7_ (based on RT-PCR data)
*K*_1_	2.1	Weighted factor
*K*_4_	11.0	Weighted factor
*K*_61_	8.6	Weighted factor
*K*_66_	1.3	Weighted factor
*K*_7_	1.2	Weighted factor
*k*_12_	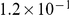	Threshold of *x*_2_ to inhibit *x*_1_
*k*_23_	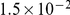	Threshold of *x*_3_ to activate *x*_2_
*k*_41_	1.7	Threshold of *x*_1_ to activate *x*_4_
*k*_61_	1.4	Threshold of *x*_1_ to activate *x*_6_
*k*_66_	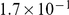	Threshold of *x*_6_ to activate *x*_6_ (auto-regulation)
*k*_65_	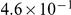	Threshold of *x*_5_ to activate *x*_6_
*k*_76_	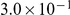	Threshold of *x*_6_ to activate *x*_7_
*n*_12_	4	Coefficient of nonlinearity for *x*_2_ to inhibit *x*_1_
*n*_23_	4	Coefficient of nonlinearity for *x*_3_ to activate *x*_2_
*n*_41_	4	Coefficient of nonlinearity for *x*_1_ to activate *x*_4_
*n*_61_	4	Coefficient of nonlinearity for *x*_1_ to activate *x*_6_
*n*_66_	4	Coefficient of nonlinearity for *x*_6_ to activate *x*_6_
*n*_65_	4	Coefficient of nonlinearity for *x*_5_ to activate *x*_6_
*n*_76_	4	Coefficient of nonlinearity for *x*_6_ to activate *x*_7_

Parameters such as mRNA degradation rates are estimated based on our data or from existing literatures. Values of other parameters such as activation/inhibition threshold *k* and relative weights *K* are estimated to fit in the time course data by genetic algorithm of least square regression. Degradation rates are in the unit of/min. Activation/inhibition thresholds, nonlinearity coefficients and relative weights for multiple inputs are non-dimensionalized.

As is shown in Figure 7B, our computational model based on the proposed signaling circuitry fits well with the experimental data. The model reveals that the essential requirement of the biphasic dynamics of C/EBPδ and LCN2 is the proper timing of the two consecutive modules. The immediate yet transient induction in c-Jun explains the first pulse in C/EBPδ, when the early-phase module dominates the dynamics of the system. As soon as c-Jun decays, the late-phase module relays the signal and an auto-regulation of C/EBPδ gives rise to the persistent induction of both C/EBPδ and LCN2. IKKε plays an important role in ensuring the correct timing of the second module. Experimentally, it was shown that IKKε activates the activity of C/EBPδ, therefore the auto-regulation of C/EBPδ is IKKε-dependent (21).

Taken together, our computational simulation based on the intracellular signaling circuitry identified in this report fitted well with the experimental data, capturing the dynamic intracellular circuits that give rise to transient induction of TNFα and persistent induction of LCN2.

## Discussion

In this report, we have identified that LPS can induce a persistent expression of LCN2 in kidney fibroblasts. The intracellular signaling circuit responsible for the persistent induction of LCN2 involves dynamic interaction among AP-1 and C/EBPδ. An early phase of a transient AP-1 activation helps to usher in a late phase of C/EBPδ activation. IRAK-1 is responsible for the propagation of both phases necessary for the persistent induction of LCN2.

Our study shed light on the dynamic and functional circuitry within cells when challenged with bacterial endotoxin. Although empirical knowledge reckons that integrated intracellular circuits instead of linear cascades are more capable of interpreting and responding properly to environmental cues including LPS, almost all existing studies have adopted conventional approaches in defining the signaling cascades downstream of LPS and TLR4 receptors [24]. To this regard, a large number of signaling molecules and pathways have been discovered that can be activated by LPS [25]. However, how these signals are properly integrated and give rise to distinct cellular responses is not clear. This work builds upon conventional information uncovered in the area of LPS signaling within host cells, and reveals an integrated circuit that couples a transient activation of AP-1 with a sustained activation of C/EBPδ.

Our effort is made possible by the integrated approach combining experimental tests and computational analyses. Taken into consideration of the experimental data, we have employed a differential equation-based approach to analyze time-dependent activation of AP-1 and C/EBPδ, as well as expression of *Tnfα* and *Lcn2*. As a consequence, genes primarily under the control of AP-1 such as *Tnfα* experience a transient pattern of expression. The expression of *C/ebpδ*, on the other hand, is initially activated by AP-1, and subsequently sustained by its own auto-activation [17], [21]. Due to the transient nature of AP-1 activation, we observed a transient dip in the expression of *C/ebpδ* (around the 4 hr time point). Subsequent self-activation of C/EBPδ then sustains the continued expression of *C/ebpδ* and its downstream gene *Lcn2*. A detailed computational analysis can be seen in the supplementary material.

It is likely that there will be significant perturbations as well as additional connections surrounding this circuit in different cell types. Consequently, this may allow distinct outcomes in different cell types in response to bacterial endotoxin. For example, the expression of C/EBPδ in macrophages induced by LPS eventually subsides, due to a novel negative feedback initiated by ATF3 [17]. In contrast, we observed that LPS failed to induce *Atf3* in kidney fibroblasts, which may explain the sustained expression and activation of C/EBPδ. This may due to the fact that fibroblasts are less sensitive to the LPS challenge as compared to macrophages. Indeed, our recent study indicates that a much lower LPS dose (∼50 pg/ml) induces a persistent activation of C/EBPδ in macrophages [26], similar to the effect seen in this study using fibroblasts with a high dose LPS. Further supporting this claim, we observed that IRAK-1 is relatively stable in kidney fibroblasts treated with 100 ng/ml LPS. In contrast, it was observed that 100 ng/ml LPS causes IRAK-1 degradation in macrophages, which can dampen the signals leading to C/EBPδ activation, leading to LPS tolerance [27]. Further studies employing systems biology approach are clearly warranted to clarify the differential sensing mechanisms of LPS signal strength in different cell types. On a separate note, the integrated systems approach will likely explain the complex phenotypes such as LPS priming and tolerance exhibited in cells treated with either a low or a high dose LPS.

Our findings bear significant clinical implication during the pathogenesis of chronic and persistent inflammatory diseases. Local tissues and fibroblasts encountering bacterial endotoxin may exhibit persistent activation of the C/EBPδ circuit without a dampening mechanism. As a consequence, inflammatory mediators such as LCN2 may accumulate and lead to chronic tissue damages including kidney failure. Strategies targeting at this functional circuit may hold promise in designing therapeutic strategies to combat chronic inflammatory diseases.
